# Temporary and highly variable recovery of neuromuscular dysfunction by electrical stimulation in the follow-up of acute critical illness neuromyopathy: a pilot study

**DOI:** 10.1186/s42466-023-00293-1

**Published:** 2023-12-28

**Authors:** Madona Sekhniashvili, Petra Baum, Klaus V. Toyka

**Affiliations:** 1 Department of Neurology, University of Leipzig, Liebigstr. 20, 04103 Leipzig, Germany; 2 Department of Neurology of S. Khechinashvili University Clinic, Tbilisi State Medical University, Chavchavadze Ave. 33, 0179 Tbilisi, Georgia; 3 Department of Neurology, University of Würzburg, Josef Schneider Str.11, 97080 Würzburg, Germany

**Keywords:** Sepsis, Critical illness neuromyopathy, Electrical stimulation, Facilitation, Nerve conduction studies

## Abstract

**Background:**

In sepsis-associated critical illness neuromyopathy (CIPNM) serial electrical stimulation of motor nerves induces a short-lived temporary recovery of compound muscle action potentials (CMAPs) termed facilitation phenomenon (FP). This technique is different from other stimulation techniques published. The identification of FP suggests a major functional component in acute CIPNM.

**Methods:**

From our previous study cohort of 18 intensive care unit patients with sepsis associated CIPNM showing profound muscle weakness and low or missing CMAPs on nerve conduction studies, six patients with different severity could be followed. In a pilot sub-study we analyzed the variability of FP during follow up. Over up to 6 weeks we performed 2–6 nerve conduction studies with our novel stimulation paradigm. Motor nerves were stimulated at 0.2–0.5 Hz with 60–100 mA at 0.2–0.5 ms duration, and CMAP responses were recorded. Standard motor nerve conduction velocities (NCV) could be done when utilizing facilitated CMAPs. Needle electromyography was checked once for spontaneous activity to discover potential denervation and muscle fiber degeneration. Serum electrolytes were checked before any examination and corrected if abnormal.

**Results:**

In all six patients a striking variability in the magnitude and pattern of FP could be observed at each examination in the same and in different motor nerves over time. With the first stimulus most CMAPs were below 0.1 mV or absent. With slow serial pulses CMAPs could gradually recover with normal shape and near normal amplitudes. With facilitated CMAPs NCV measurements revealed low normal values. With improvement of muscle weakness subsequent tests revealed larger first CMAP amplitudes and smaller magnitudes of FP. Needle EMG showed occasional spontaneous activity in the tibialis anterior muscle.

**Conclusion:**

In this pilot study striking variability and magnitude of FP during follow-up was a reproducible feature indicating major fluctuations of neuromuscular excitability that may improve during follow-up. FP can be assessed by generally available electrophysiological techniques, even before patients could be tested for muscle strength. Large scale prospective studies of the facilitation phenomenon in CIPNM with or without sepsis are needed to define diagnostic specificity and to better understand the still enigmatic pathophysiology.

*Trial registration*: This trial was registered at the Leipzig University Medical Center in 2021 after approval by the Ethics Committee.

**Supplementary Information:**

The online version contains supplementary material available at 10.1186/s42466-023-00293-1.

## Background

Critical illness polyneuromyopathy (CIPNM or CIP/CIM) is a common neuromuscular complication in sepsis patients on intensive care units (ICU). CIPNM leads to mechanical ventilation and prolonged weaning [1, 2]. Over some decades critical illness polyneuropathy (CIP) and critical illness myopathy (CIM) have been maintained as separate entities and investigated as such [3–5]. Others proposed a mix of both in the majority of cases and some proposed a dominant role of CIM [2, 6–9]. The pathogenesis of CIPNM remains incompletely understood [2, 8, 10]. Electrodiagnostic nerve conduction studies (NCS) in CIPNM revealed small or absent compound muscle action potentials (CMAPs) in the acute phase and sensory nerve action potentials alike (SNAPs). With absent CMAPs and SNAPs nerve conduction velocities could not be measured [6, 11]. Spontaneous activity was found in muscles on electromyography in some patients [10]. However, all standard NCS showed unspecific abnormalities in patients with CIPNM. Moreover, they could not differentiate CIPNM from other axonal or mixed neuropathies, nor from other types of myopathies [6, 10–12]. Ultrasound may discover nerve swellings in CIPNM but again, this is not specific for CIPNM [13]. Muscle biopsies are not generally accepted as a diagnostic tool in these severely ill patients with sepsis and may not be widely available in contrast to standard NCS [8]. In another study in patients [14], and in a rat model [7], peripheral nerve and muscle fibers both showed a reduced excitability with inactivation of sodium channels and this was proposed as the principal cause of the weakness in CIPNM. In a more recent study on ICU patients defined as CIP inactivation of voltage-gated sodium channels was proposed as a prime disease mechanism [15]. In another study the dominant role of the myopathic contribution in CIPNM patients was identified by quantitative electromyography, direct muscle stimulation and NCS [9].

Recently we detected a novel electrophysiological feature in ICU patients clinically diagnosed with sepsis associated CIPNM. With slow serial electrical stimuli at 0.2–0.5 Hz to motor nerves the initially absent or very small CMAPs showed a marked and often exponential gradual rise of motor responses temporarily, and this pattern was termed facilitation phenomenon (FP) [16]. FP was also observed by direct non-invasive muscle serial stimulation in the tibialis anterior muscle. These findings were interpreted as conditioning effects causing a partial reversal of neuromuscular dysfunction. Sepsis patients with equal severity as measured by clinical sepsis scores but without muscle weakness did not show absent first CMAP amplitudes nor FP at 0.3–0.5 Hz stimulation [16]. In addition, a heterogeneous neuropathy control group with abnormal NCS was tested without showing FP [16].

Here we report about six patients from that published cohort, in whom we were able to do repeated motor nerve stimulation tests for up to 6 weeks, allowing us to investigate variability of FP and to perform a new variant of NCS utilizing facilitated CMAPs.

## Methods

### Patients

At the ICUs of the Interdisciplinary Internal Medicine Intensive Care Division at the Leipzig University Medical Center we studied six patients with sepsis associated CIPNM repeatedly during follow-up for a various length of time (Table 1a and b). The only selection criterion of these six patients was the ability to explore their FP over time. This was a very first attempt exploring reproducibility and variability of FP-positive patients with different clinical severity of CIPNM. No patient had a history of identified pre-existing neuromuscular disorders including myasthenia gravis, Lambert-Eaton syndrome, overt polyneuropathy, polytrauma, neurotoxic or high-dose corticosteroid treatments were not part of the history, and none had a positive SARS-Co-V2 PCR test or received neuromuscular blocking agents at the time of testing. Five of the six patients were clinically defined as CIPNM by an abnormal Medical Research Council (MRC) sum score [17] of < 48/60 as soon as they could be formally examined. Only one of the six patients (patient 1) could not be formally tested for muscle weakness with MRC scoring but elicited reflex movements indicated profound weakness throughout the entire observation period until his death. Short individual case reports are described in Results. Five of the six patients had septic shock with multiple organ failure, while one (patient 6) was less severely afflicted from sepsis. We did not re-analyze sepsis patients of our control group without muscle weakness who did not show FP as reported [16].

### Electrophysiological examinations

Awake and cooperative patients were informed about indication and technique of the procedures and gave their consent. If patients were not able to give informed consent, the next of kin or a custodian was asked for permission, according to the approved protocol of the Ethics Committee at the University Medical Center at Leipzig. In the four surviving patients informed individual consent was confirmed by the patients when becoming cooperative. No patient had a nerve or muscle biopsy. Patients had daily laboratory investigations. In case of electrolyte imbalances these were corrected before the examination. All examinations were done at the bedside utilizing a Windows 10-based laptop digital EMG machine (Neurosoft NET-Omega software, Evidence 3201, Schreiber&Tholen, Stade, Germany). Filter settings were 5 Hz and 10,000 Hz, 50 Hz notch filter, and the post-stimulus artifact suppression was set at 0.6 ms.

### Motor nerve conduction studies and the facilitation phenomenon (FP)

In the six patients FP of the motor responses were studied during follow-up as described [16]. In brief, the distal tibial nerve was stimulated bilaterally and the peroneal nerve unilaterally, distally and proximally (Table 1a and b). In one patient the ulnar nerve was also examined. All patients had initially absent or very low distal CMAPs preventing standard nerve conduction studies. The number of identical stimuli per series was 10–15 depending on the patient’s tolerance. Pain tolerance of the patient and accessibility at a given time limited the strict adherence to our standard electrophysiological protocol. For the six patients tests were repeated at the same day and at different days during follow-up, whenever this was feasible and tolerated by the patients. There were no consistent predetermined examination time points on follow-up in this exploratory investigation. This was due to limitations in accessibility of nerves and in pain tolerance of the patients. It also depended on the availability of patients for a repeated examination at a given time point. Therefore, we could not do the serial stimulations in the same nerves at every time point. In the published cohort of 18 ICU patients (16) as part the main study, a sepsis control group was included and tested under the identical conditions showing normal CMAP amplitudes and no FP when applying the serial stimulation paradigm. For the present pilot study we did not see a justification for examining more sepsis patients without muscle weakness. Each patient was his own control for the evaluation of variability.

Motor responses were recorded from the abductor hallucis muscle when stimulating the distal tibial nerve, from the extensor digitorum brevis muscle when stimulating the peroneal nerve, both distal and proximal, and from the abductor digiti minimi muscle for the ulnar nerve. Self-adhesive tape electrodes were used as recording electrodes after alcohol cleaning of the skin throughout. Electrodes were left exactly in the same place until the next test was done up to a few hours later. With longer time intervals a new set of tape electrodes was placed at the identical positions. Since the same experienced examiner (MS) was doing all the tests this procedure was considered appropriate. All nerves were tested with square wave pulses at a slow frequency of one stimulus, every 2–5 s (0.2–0.5 Hz) and with a standard current of 100 mA in the legs and 60 mA in the arm, and with 0.2 ms pulse duration. Occasionally 0.5 ms had to be used if no discernible CMAPs appeared. In two patients 1 Hz trains were used once with 0.5 ms pulses. No formal stepwise evaluation of maximal and supramaximal currents was done to avoid pre-conditioning before the full series of stimuli was applied. The initial and the maximal CMAP amplitudes were recorded in each examined muscle upon a series of electrical pulses and stored for further calculations. The absolute gain in CMAP amplitudes, from the lowest (minimal) to the highest (maximal) CMAP, was calculated in the first and at any subsequent examination (first and last are listed in Table 1a and b). In addition, the different patterns of variability were categorically defined and listed in Table 2. In all recordings, the negative peak durations of the 1st discernible CMAP and of the last facilitated CMAP were used for calculations.

Because of the absent or very small 1st CMAPs we could not estimate distal motor latencies in 5/6 patients for the peroneal and in 4/6 for the tibial nerve. Only facilitated CMAPs allowed us using the new technical variant of NCS in this pilot study. This procedure had not yet been done in the reported study (16). With maximal CMAPs (FP -max in Table 1a and b) we could analyze the shape of the CMAPs and measure distal latencies and NCVs in the peroneal, tibial or ulnar nerve (Table 1a and b), as it is done in standard nerve conduction testing. As described before the eight neuropathy control patients showed no FP (16).

Needle EMG tests were performed once during the follow-up in the tibialis anterior muscle with standard coaxial needle electrodes: in 4/6 about 14 days after the presumed CIPNM onset and in 2/6 at an earlier time point (Table 1a and b). The examination was restricted to identifying spontaneous activity (SpA) as a marker of nerve or muscle fiber degeneration. Quantitative EMG could not be done, due to lack of patient cooperation.

Statistical evaluations were not feasible in this exploratory pilot study.

## Results

### Clinical data

Patients were examined electrophysiologically and clinically up to 6 times at the ICU or after discharge to another unit.

#### Patient 1

The 58 years old male was found comatose with generalized convulsions and hypothermia. After cardiopulmonary resuscitation circulation was restored. Septic shock ensued with multiple organ failure and pneumonia requiring invasive ventilation. Continuous dialysis was needed. The patient developed severe disseminated coagulopathy with ischemic defects in brain stem and lower legs. Weaning from the respirator remained unsuccessful. Vigilance remained poor and formal MRC muscle testing was impossible. Involuntary and induced reflex movements were all weak. The clinical diagnosis of CIPNM was made 7 days after onset of septic shock. The patient deceased 2 weeks later.

#### Patient 2

The 50 years old male was admitted to the ICU with severe respiratory insufficiency, in need of continuous invasive ventilation. He had a COVID-19 infection 1 month before and pulmonary fibrosis, and the PCR-test became negative. Septic shock, renal failure requiring dialysis, and transient delirium were diagnosed. Pulmonary gas exchange remained insufficient requiring oxygenation. Weaning attempts were unsuccessful. The approximated MRC sum score was 30/60 confirming CIPNM. The patient deceased after 42 days.

#### Patient 3

The 42 years old male suffered from cardiopulmonary arrest during a severe asthma attack and was successfully resuscitated within 10 min. On admission at the ICU sepsis was diagnosed with multiple organ failure, pneumonia, and urinary tract infection with preserved kidney function. EEG showed a non-convulsive status epilepticus which was stopped by treatment with propofol, and this treatment was continued until the EEG did not show further epileptic discharges. The patient improved gradually in vigilance and cognition with no further seizures, and muscle strength could be formally tested with a MRC sum score of 32/60 clinically confirming CIPNM. Weaning was possible after 29 days. During follow-up muscle strength slowly improved.

#### Patient 4

The 79 years old male was admitted to the ICU with pneumonia, encephalopathy, septic shock, and renal failure requiring short term dialysis treatment. Invasive ventilation was not needed. On day 3 the MRC sum score revealed 48/60 clinically confirming CIPNM. He improved and signs of sepsis remitted. On day 8 the MRC sum score was 56/60.

#### Patient 5

The 66 years old female had a chronic obstructive pulmonary disorder and rheumatoid arthritis treated with low dose prednisolone (up to 10 mg/d). She acquired acute pneumonia with sepsis, global respiratory and cardiac failure, and with encephalopathy requiring invasive ventilation, for a total of 6 days. The MRC sum score was 48/60 at day 4 after sepsis onset clinically confirming CIPNM. The patient improved rapidly and could be discharged within 16 days with a MRC sum score of 58/60.

#### Patient 6

The 76 years old female with moderate Parkinson’s disease was admitted to the ICU with pneumonia and urinary tract infection resulting in sepsis. Invasive ventilation was needed for 1 day only. Vigilance improved and the MRC sum score was 44/60 on day 2 which clinically confirmed CIPNM. The patient improved over the next 3 days and was transferred to a regular ward. At day 44 she had a normal MRC.

### Electrophysiological findings

Follow-up FP examinations were done over a period of 5–44 days after sepsis onset. In Table 1a and b the data of the initial and the last electrophysiological examinations are listed. All patients showed the facilitation phenomenon (FP). The following results were found:

#### Huge variability in the reproducibility and in the various patterns of FP

**Fig. 1:**
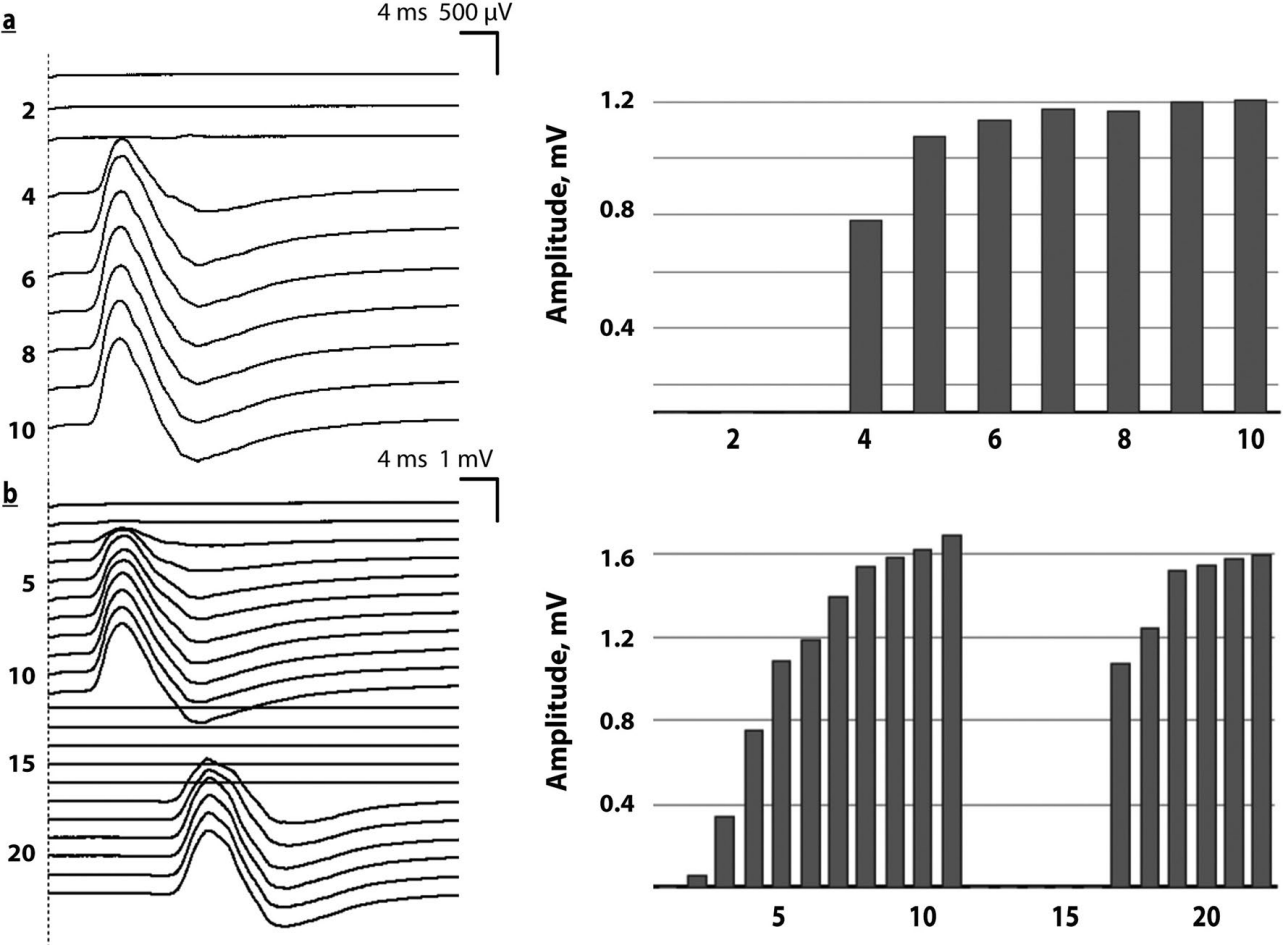
Variability of peroneal motor nerve induced excitability with early serial stimulation. Patient 1: day 2 after sepsis onset: on left: original recordings. **a** distal: 1st CMAP with the 4th stimulus; moderate FP, low maximal CMAP amplitudes **b** 45 min later: delayed 1st CMAP from distal and proximal stimulation followed by FP. Vertical scale: sequence of stimuli; on right: bargraphs showing CMAP amplitudes at the vertical scale; horizontal scale: sequence of stimuli applied; note that facilitated maximal CMAP amplitudes remain abnormally low

**Fig. 2:**
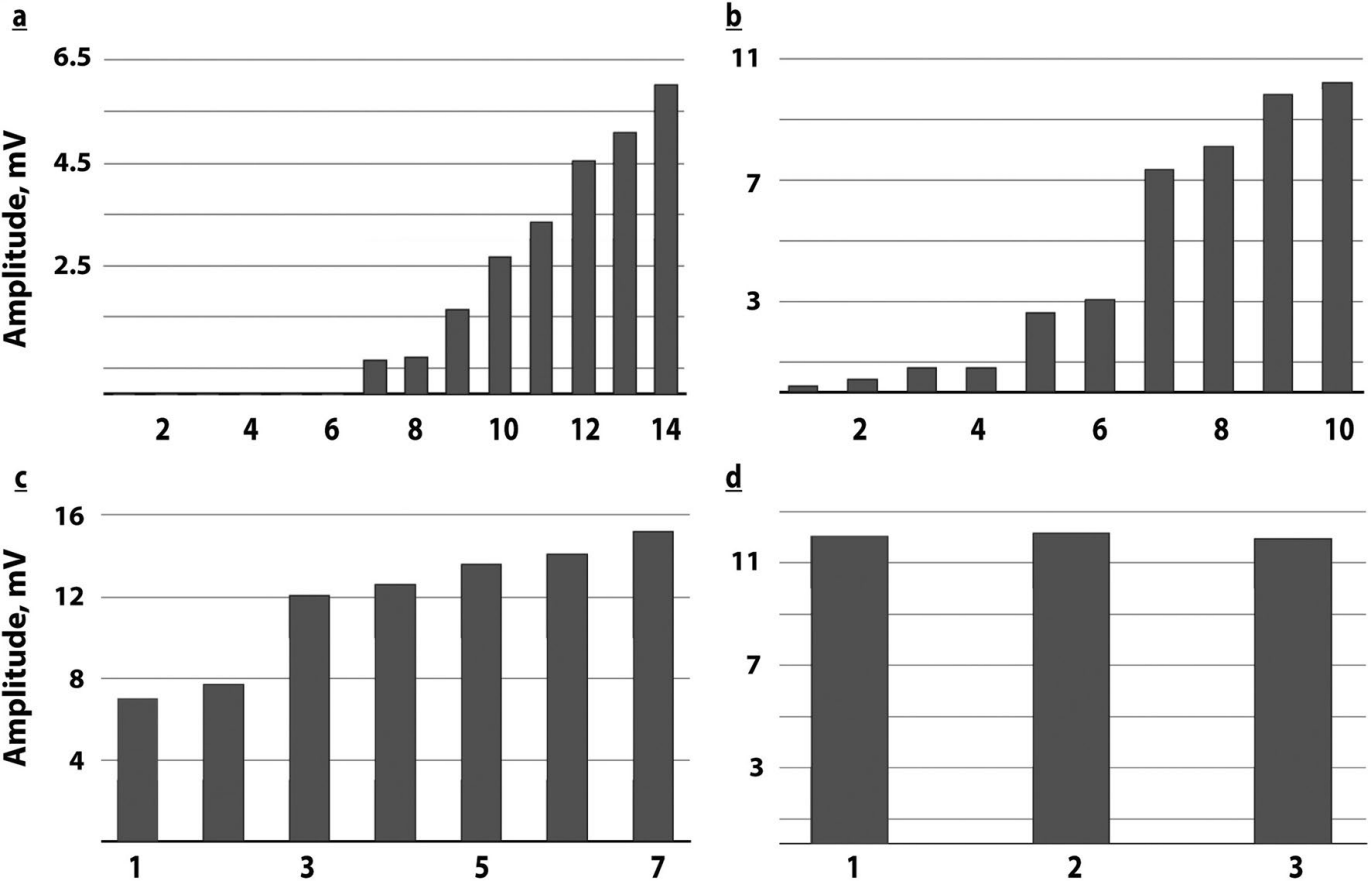
Long-term variability of tibial motor nerve induced excitability with serial stimulation. Patient 6: days 2 and 36 after sepsis onset- serial distal stimulation of the left (upper panel) and right (lower panel) tibial nerves: day 2: **a** left tibial: 6 stimuli induce isoelectric lines, 1st CMAP appears with 7th pulse and FP is shown reaching normal CMAP amplitude; **b** right tibial: immediate small 1st CMAP is evoked, FP ensues with higher maximal CMAP amplitudes than in **a**; day 36: major clinical improvement: **c** left tibial: FP is still present with much higher initial and higher maximal CMAP amplitudes; **d** right tibial: no more FP, normal 1st CMAP amplitude. Bargraphs illustrate gain in amplitudes; Vertical scale: amplitudes of the original recordings. Horizontal scale: sequence of stimuli applied

**Fig. 3:**
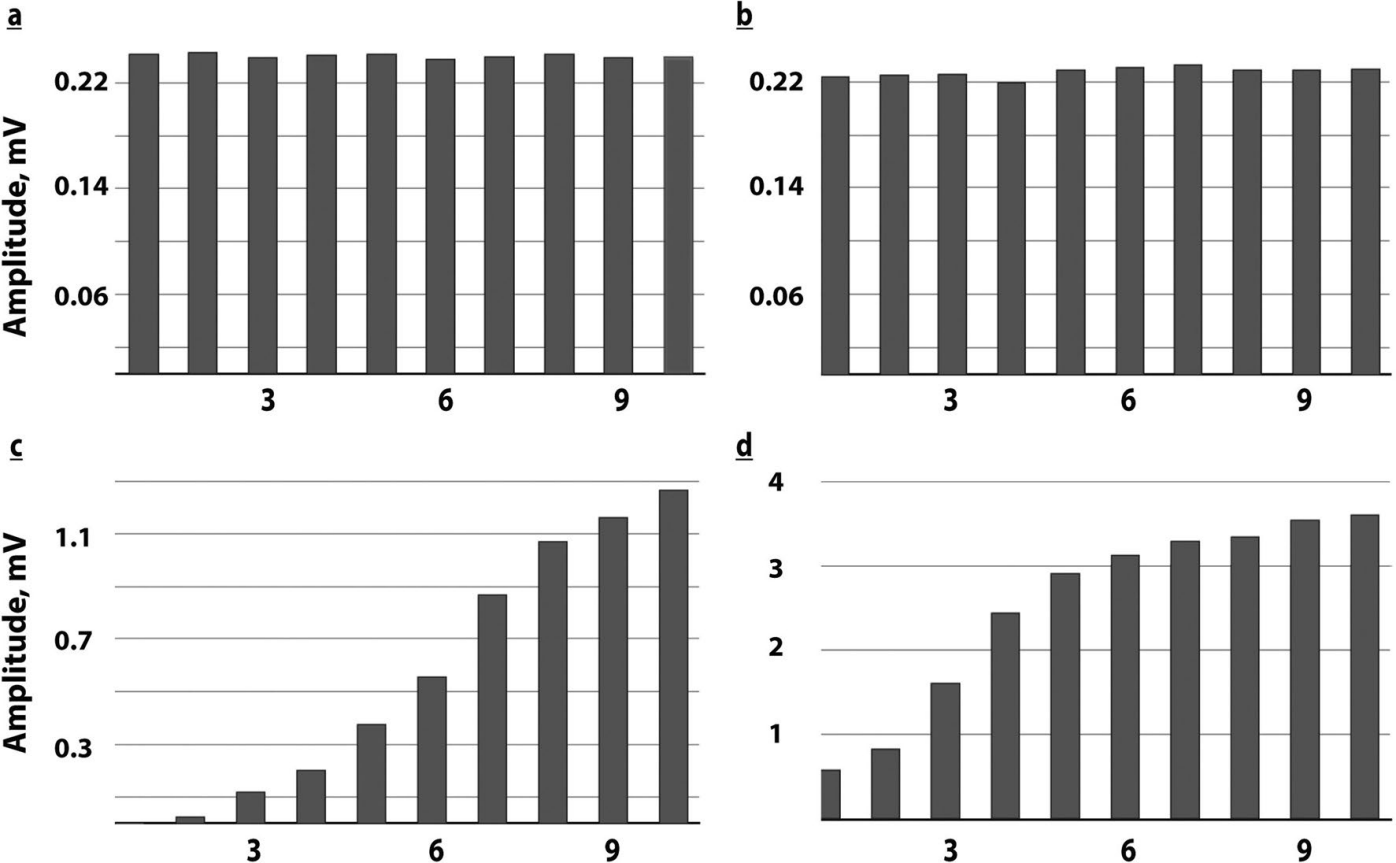
Short-term variability of tibial and peroneal motor nerve induced excitability with serial stimulation. patient 1:day 7 after sepsis onset-; repeated series of distal stimulation of the peroneal (upper panel) and tibial nerves (lower panel), only 6 min apart, showing short-term FP variability, on right (**a**) and on left (**b**) the peroneal nerve induces abnormally low CMAPs without FP; both tibial nerves induce FP: **c** 1st CMAP with 2nd stimulus at the right tibial nerve showing still abnormally low maximal CMAPs; **d** now an immediate 1st CMAP appears; maximal CMAP amplitudes are induced by stimulating the left tibial nerve reaching normal range; note the different shape of the FP pattern when compared to **c**. Bargraphs: amplitudes of the original recordings. Horizontal scale: sequence of stimuli applied

**Fig. 4:**
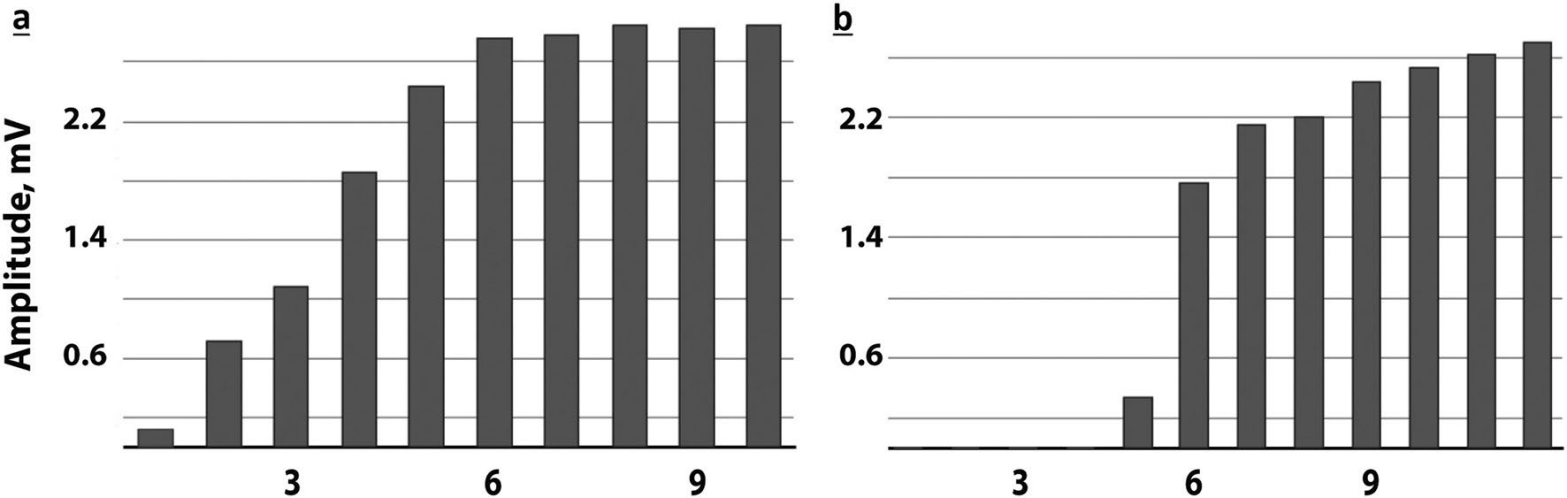
Variability of ulnar motor nerve induced long-term excitability with serial stimulation. Patient 3, right ulnar nerve **a** day 12 after sepsis onset: small 1st CMAP with FP **b** day 17: delayed 1st CMAP and different shape of FP; maximal CMAP amplitudes remain below normal limits in both recordings. Bargraphs: amplitudes of the original recordings. Horizontal scale: sequence of stimuli applied

The severe excitability defects that could be reversed temporarily through serial stimulation were reproduced at each follow-up examination, but at a highly variable degree. The magnitude and types of variability changed over minutes, an hour, or a couple of days. The stimulation of the distal or proximal segments of the identical peroneal nerve could elicit variable patterns and magnitudes of FP. The same was also observed in the tibial nerve, where only distal segments were tested in 5/6 patients. Representative examples illustrating variability patterns are shown (Figs. 1, 2, 3, 4). We named these patterns using categorial terms (Table 2). During follow-up most of these patterns could be seen in any of the examinations but often in different combinations. All these features corroborate our interpretation of the findings as indicating highly fluctuating functional defects in CIPNM.

**Table 1:** Summary of electrophysiological findings

(a)						
Pat no	1st FP/DFSO	1st min CMAP Per/Tib (mV)	FP-max CMAP Per/-Tib (mV)	FP-max CMAP Dur. Tib (ms)	FP-maxDML Per/-Tib (ms)	FP-NCV m/s stim. Nerve
1	*14*	< 0.01 < 0.01	0.18 3.4	5.3	2.8 4.0	48 Tib
2	*7*	0.24 < 0.01	0.8 2.7	9.2	3.8 3.9	40 Per
3	*12*	0.01 < 0.01	0.12 4.9	5.2	3.1 3.0	41 Per
4	*3*	0.11 0.02	1.1 1.2	5.5	3.4 3.4	52 Per
5	*2*	< 0.01 0.7	1 4.2	5.2	3.3 3.2*	45 Uln
6	*2*	0.01 0.18	3.1 10.2	5.6	2.8 3.5	nd

(b)							
Pat no	Days after 1st FP	1st min CMAP Per/Tib (mV)	FP-max CMAP Per/-Tib (mV)	FP- max CMAP Dur Tib (ms)	FP-DML Per/ Tib (ms)	Outco-me/DFSO	SpA on EMG
1	*7*	0.21 < 0.01	0.37 1.6	5.2	4.4 3.6	28**†**	PSW+
2	*32*	0.21 0.9	0.21 3.8	7.4	5.1 5.7	42**†**	None
3	*25*	0.14 < 0.01	n.d 3.8*	5.4	2.8 3.6	37++	Fibs+
4	*5*	0.01 2.5	0.16 3.7	7.2	4.2 3.6	6+++	None
5	*6*	n.d 8.9	n.d 9.2	5.3	n.d 3.2	16++	None
6	*44*	2.3 7	2.5 15.2	3.8	3.6 3.2	9+++	None

(a): The data obtained at the first electrophysiological examination are shown: 1st column: patient number, 2nd column FP DFSO with number of days from sepsis onset until FP was first examined; 3rd column: 1st minimal CMAP amplitude at the indicated nerves before serial stimulation; 4th column: maximal CMAP amplitudes upon serial stimulation at the indicated nerves; 5th column: duration of maximal CMAPs at the indicated nerves; 6th column: distal motor latencies obtained with maximal CMAPs - peroneal and tibial nerves; 7th column: Nerve conduction velocities obtained with maximal CMAPs at the indicated nerves

(b): All data are from the last follow-up examination: 1st column: patient number; 2nd column: days after the 1st electrophysiological examination; 3rd column: minimal CMAP amplitude at the indicated nerves before serial stimulation; 4th column: maximal CMAP amplitudes upon serial stimulation at the indicated nerves; 5th column: duration of maximal CMAPs at the indicated nerves; 6th column: distal motor latencies obtained with maximal CMAPs – peroneal and tibial nerves; 7th column: clinical outcome; 8th column spontaneous activity obtained with needle EMG

*Fibs* fibrillation potentials, * here only 3 serial stimuli could be applied, *FP-DML* distal motor latency as measured with facilitated max CMAPs, *FP-max CMAP* maximal CMAP amplitude reached during FP, *FP-NCV* nerve conduction was calculated from distal and proximal facilitated maximal CMAPs in the indicated nerves, *n.d.* not done, *Per* peroneal nerve values (listed below the tibial nerve values); needle EMG, *PSW* positive sharp waves, *SpA* spontaneous activity, *Tib* tibial nerve values listed (above peroneal values), *Uln* ulnar nerve; Outcome at day indicated: +; ++; +++ mildly-moderately-markedly improved muscle weakness. Patients 1 and 2 deceased. For normal values see Additional file 3

**Table 2:** Patterns of variability

Pattern #	Definition of patterns of variability
1	Variability of FP on left versus right in the same nerve
2	Variability in the shape of FP between different nerves
3	Variability in the shape of FP at the same nerve and side over time
4	Variability of FP when serially stimulating distally versus proximally
5	Variability in the number of stimuli evoking a 1st CMAP
6	Variability in the number of stimuli to reach highest CMAP

Six different patterns of variability were found that were superimposed in various combinations

#### Nerve conduction studies as measured with facilitated CMAPs showed no major abnormality

When the initially absent or very small CMAPs started to rise upon serial electrical stimulation, the facilitated biphasic CMAPs looked normal and could even reach normal amplitudes. With the initially very low or absent 1st CMAPs (Table 1a on left) the distal motor latencies were not obtainable in 4/6 tibial and in 5/6 peroneal nerves and no conduction velocities could be analyzed in the peroneal nerves in any of the six patients. Only when utilizing facilitated CMAPs, we could do NCS with measuring distal motor latencies in the tibial and peroneal nerves. These were within the normal range in all 6/6 patients (Table 1a). Motor NCVs could be done in the peroneal nerve in 3/6, in the tibial nerve in 1/6, and in the ulnar nerve in 1/6 patients, and conduction velocity values were found to be in the normal or lower borderline range (Table 1a). During follow-up there appeared neither CMAP dispersion nor conduction block in the 3/6 patients tested for peroneal motor NCV (Fig. 1), or in the 2/6 patients, one with tibial NCS and one with ulnar NCS. (Data not shown) A technical limitation in these NCS was that the high current strength setting was predetermined when eliciting FP, without the formal stepwise evaluation of supramaximal currents.

#### Improvement of nerve-muscle dysfunction may go along with clinical amelioration

In the patients with a good clinical recovery (Table 1b, patients 4, 5, and 6), the minimal and maximal CMAP amplitudes, within a series of stimuli, became higher and the calculated FP ratios became smaller on follow-up (Table 1a and b, Fig. 2). In CIPNM patients 1 and 2 who eventually deceased, we observed extremely variable patterns and magnitudes of FP at several examinations (Figs. 1, 3). Only in these two patients we could observe a temporary state of complete loss of excitability after a previous series had induced FP (data not shown).

#### FP elicited with 1 Hz stimulation

In patients 1 and 2 we once used a 1 Hz series of stimulation, in addition to the slower series minutes before (Additional file 1: Figure S1). FP could also be elicited at I Hz.

#### Variable reduction of CMAP duration during FP

When FP was generated the durations of the negative peaks of the 1st discernible CMAP and of the 10th CMAP were measured and compared. The duration of the 1st CMAP ranged from 5.0 to 10.9 ms (median 6.8). In 5/6 patients a gradual shortening of the duration was seen along the series of facilitated CMAPs. Only in 1/6 there was a marginal increase in duration. The relative differences of durations were calculated ranging from + 4 to − 27% (median—16%) in individual CIPNM patients. The shape and the duration pattern of serial CMAPs is illustrated in patients 1 and 2 (Additional file 2: Figure S2). When re-evaluating the original recordings from our published sepsis control cohort ([16], n = 7) no prolongation of duration was observed.

#### Needle EMG examinations showed little if any SpA

We found no (in 4/6 patients), very mild (in 1/6) or moderate (in 1/6) SpA comprised of rare positive sharp waves and fibrillation potentials, but no or very few fasciculations (Table 1b). In patients 1–4, with EMGs done after around 14 days, our findings indicate absent or only minor signs of degeneration.

## Discussion

In extension of the first description of the facilitation phenomenon [16], we did follow-up examinations in a sub-cohort of six sepsis associated CIPNM patients, selected by being available for follow-up. The severity of sepsis and of CIPNM was very different over the observed 2–6 weeks. The clinical spectrum included two patients with lethal disease and four who improved or even recovered. We found reproducibility and striking variability of FP with largely different patterns over time, in the same patients and even in the same nerve. The differences in FP between the tibial and peroneal nerve stimulation were remarkable, yet with an unpredictable magnitude when compared to subsequent examinations.

FP may start a few days after sepsis onset and may gradually disappear in the survivors as shown here. The reduction of the calculated FP during follow-up in patients 3 to 6, showing higher CMAP amplitudes and accordingly lesser FP, may go along with clinical improvement. We were unable to test the same nerves at each examination during follow up. This excludes direct comparison of the variability in a particular nerve over time. Due to the limited number of follow-up patients only preliminary conclusions can be drawn.

The new technical variant of measuring NCVs and distal latencies was shown to be technically feasible in CIPNM patients with initially absent CMAPs. Collectively, the NCS data obtained with facilitated CMAPs do not indicate a demyelinating or mixed neuropathy [18]

Hence, our observations challenge previous reports claiming that the commonly seen absent or very small 1st CMAPs in CIPNM indicate an axonal or mixed type of a neuropathy [4, 6, 8, 10–12].

Another reported feature of CIPNM or CIM is an increase in CMAP duration [5, 7]. These reports proposed an abnormal velocity-recovery cycle in muscle fibers as the underlying mechanism. In five of our six patients we only found a mild increase in CMAP duration in the first few CMAPs which disappeared with subsequent stimuli.

On needle electromyography there were no or only mild abnormalities in our six patients. This suggested that axon or muscle fiber degeneration was not a predominant feature at the time of examination during the acute phase of CIPNM. This is in line with findings of another recent report [11].

### Potential relevance for the pathophysiology of CIPNM

The waxing and waning excitability behavior of CMAPs in our six CIPNM patients reflects a dynamic functional defect of the neuromuscular units. There are several hypothetical disease mechanisms, none of which we could directly address in this electrophysiological study. Our present and previous observations cannot differentiate between nerve and muscle, in terms of the main afflicted organ in CIPNM. In one clinical report on CIPNM ICU patients, a reduced excitability in skeletal muscle fiber membranes was found, and this was proposed as a typical feature of CIM [19], while another study found reduced excitability in nerve axons and in muscle fibers in patients defined as CIP [10, 14]. In a recent electrophysiological study on ventilated and cooperative patients with acquired ICU weakness the author found abnormal electromyographic motor unit potentials by quantitative EMG in 33 of 35 patients [7]. This was interpreted as pointing to muscle as the prime organ afflicted. The association of CIPNM with septic shock suggests that several hypothetic pathogenic pathways could have a complementary impact on both anatomical compartments of the neuromuscular system [5, 14, 15, 20]. We speculate that the fluctuating excitability described here may reflect a rapidly changing local metabolic environment causing a reduced and highly variable blood flow and critical hypoxia in the limbs. The pathogenic final consequences may be an increased inactivation of sodium channels and a reduced excitability in nerve and muscle fibers. The latter had been shown experimentally in a rat sepsis model (7). Of note, no serial stimulations were done in any of these previously reported studies, neither in patients nor animal models.

In summary, several experimental observations appear compatible with our findings of highly variable and short-lived reversibility induced by current pulses [14] but none has provided evidence that might elucidate which of the potential mechanisms may be operative in our serial stimulation FP paradigm.

## Conclusion

This report on the variability of the recently discovered FP analyses and its changes in CIPNM over time may encourage clinical researchers to invest in larger studies with high numbers of patients. These are needed to establish specificity and sensitivity of FP as a diagnostic tool in CIPNM and may eventually help calculating a predictive value of serial FP testing for clinical outcome.

Our previous and our present study data [16] lead us to propose CIPNM as a fluctuating neuromyopathy, with no or only minor fiber degeneration in the early phase of CIPNM. There is a need for new sepsis mouse model investigations. That might allow direct comparison with our electrophysiological observations in patients and find new hints as to the pathophysiology of CIPNM (Additional file 3).

### Supplementary Information

**Additional file 1**. **Figure S1** Tibial motor nerve induced FP with 1 Hz serial stimulation. Original recordings; facilitation of CMAPs by distal stimulation of the right tibial nerve (a) patient 1: day 14 after sepsis onset, and in (b) patient 2: day 7 after sepsis onset. Vertical scale: sequence of stimuli applied in a) and b). Note that the normal biphasic shape of the facilitated CMAPs is similar to the FP series with lower frequency stimulation, with both patients (data not shown); vertical scale: sequence of stimuli applied.

**Additional file 2**. **Figure S2** Compound muscle action potential duration with serial stimulation. Original recordings; facilitation of CMAPs induced by distal serial stimulation at 0.5 Hz, rt. tibial nerve: Time markers 1 (offset) and 3 (isoelectric zero transition) indicate negative peak duration; (a) patient 1: moderate reduction of CMAP duration from 6.9 to 5.3 ms (− 23 %); (b) patient 2 mild reduction of CMAP duration from 10.9 to 9.2 ms (− 16 %). Vertical scale: sequence of stimuli applied.

**Additional file 3**. **Table S1** Normal values in motor nerve conduction studies at the institutional EMG Laboratory.

## Data Availability

The datasets used and/or analyzed during the current study are available from the corresponding author on reasonable request.

## Abbreviations

CIPNM: Critical illness polyneuromyopathy
CMAPs: Compound muscle action potentials
DML: Distal motor latency
EEG: Electroencephalography
EMG: Electromyography
FP: Facilitation phenomenon
Hz: Hertz, repetitions per second
ICU: Intensive care unit
K^+^: Potassium ions
mA: Milliampere
MRC sum score: Medical research council sum score
ms/msec: Milliseconds
Na^+^: Sodium ions
Na–K ATPase: Sodium potassium adenosine triphosphatase
NCS: Nerve conduction study
NCV: Nerve conduction velocity
