# Supplementary figures and images for: Temporary and highly variable recovery of neuromuscular dysfunction by electrical stimulation in the follow-up of acute critical illness neuromyopathy: a pilot study
Source: Neurol Res Pract. 2023 Dec 28;5:66. doi: 10.1186/s42466-023-00293-1 (PMC10753844; doi:10.1186/s42466-023-00293-1)

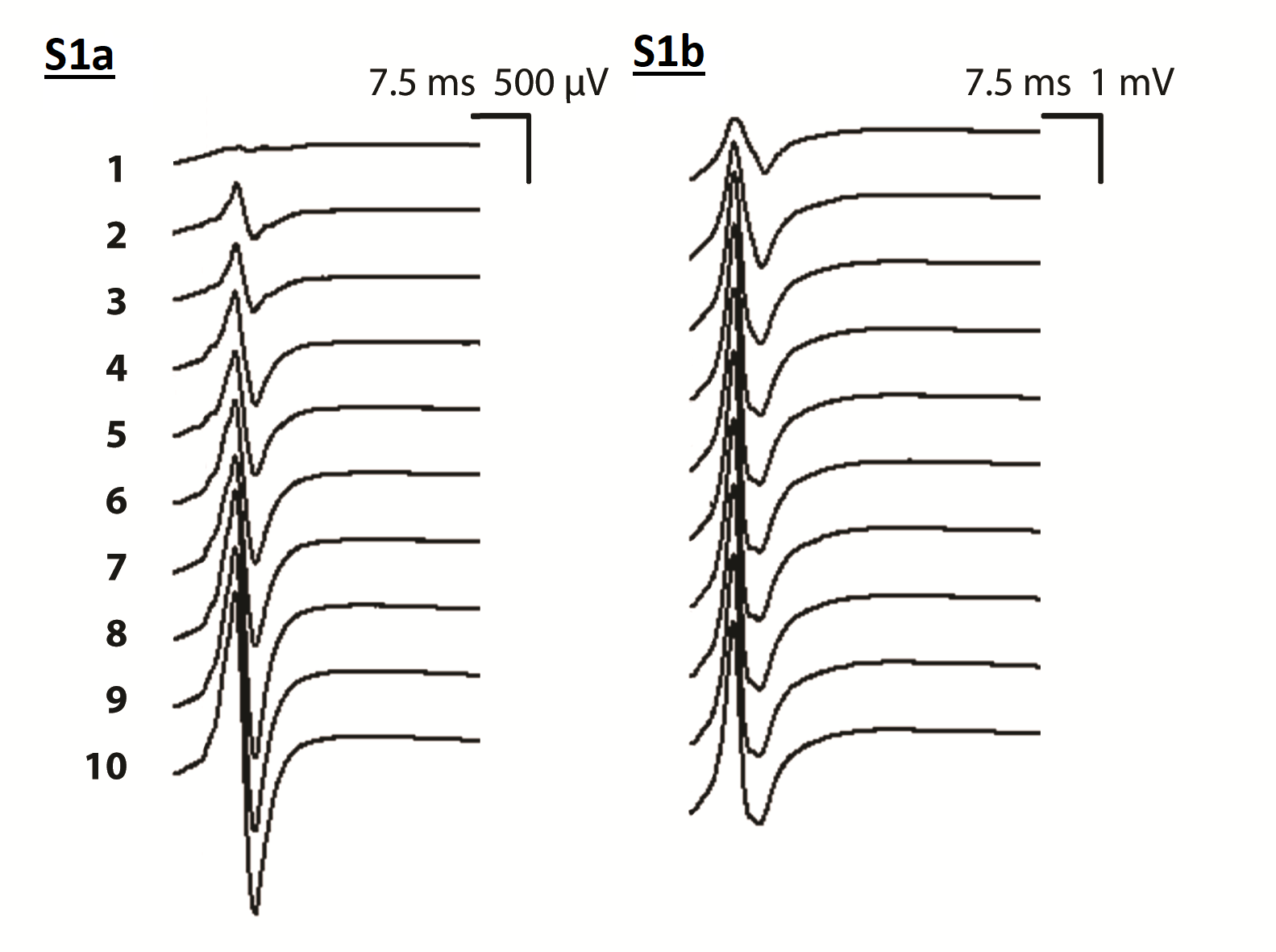

Supplement: Supplementary file 1 — Additional file 1. Figure S1 Tibial motor nerve induced FP with 1 Hz serial stimulation. Original recordings; facilitation of CMAPs by distal stimulation of the right tibial nerve (a) patient 1: day 14 after sepsis onset, and in (b) patient 2: day 7 after sepsis onset. Vertical scale: sequence of stimuli applied in a) and b). Note that the normal biphasic shape of the facilitated CMAPs is similar to the FP series with lower frequency stimulation, with both patients (data not shown); vertical scale: sequence of stimuli applied. [file 42466_2023_293_MOESM1_ESM.tif]

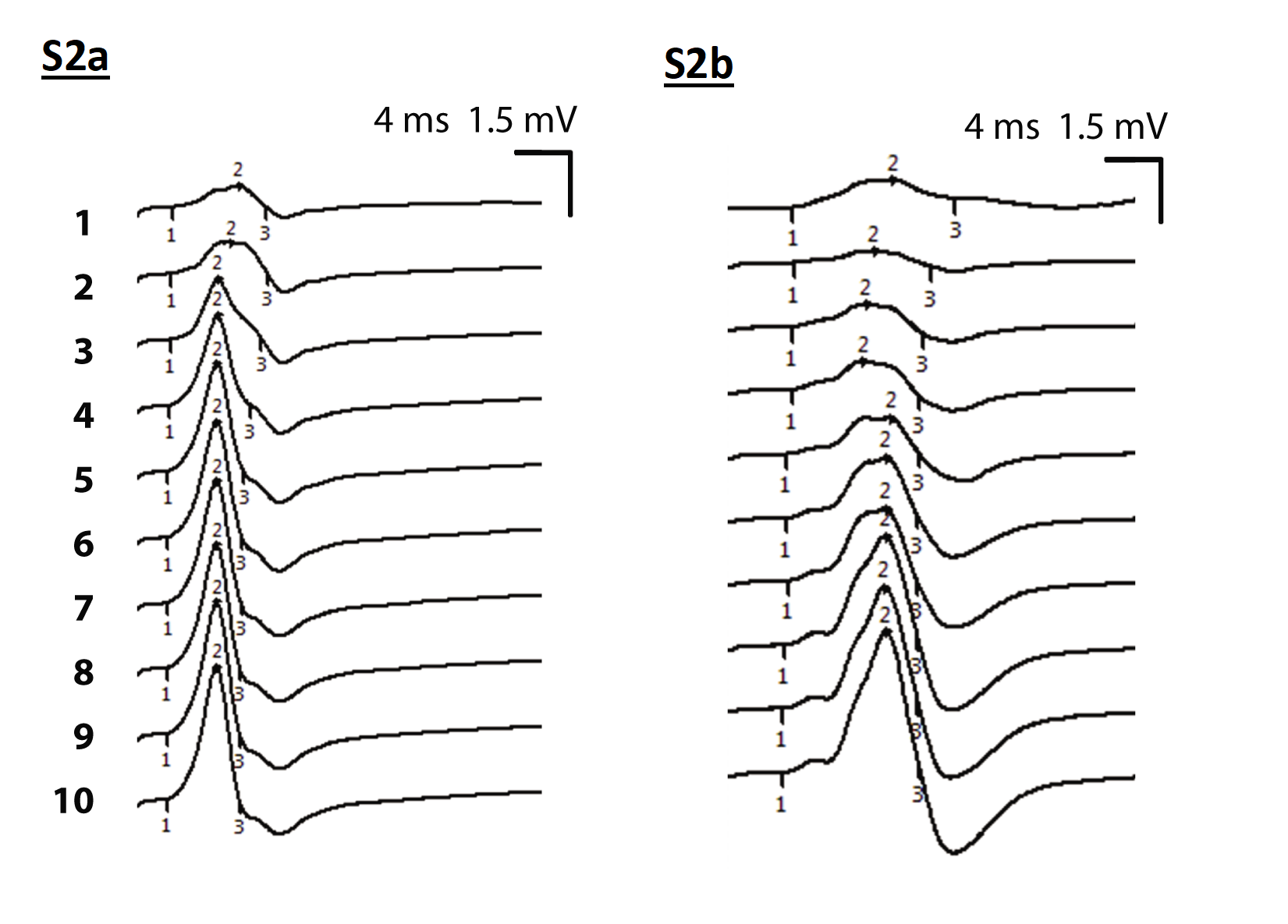

Supplement: Supplementary file 2 — Additional file 2. Figure S2 Compound muscle action potential duration with serial stimulation. Original recordings; facilitation of CMAPs induced by distal serial stimulation at 0.5 Hz, rt. tibial nerve: Time markers 1 (offset) and 3 (isoelectric zero transition) indicate negative peak duration; (a) patient 1: moderate reduction of CMAP duration from 6.9 to 5.3 ms (− 23 %); (b) patient 2 mild reduction of CMAP duration from 10.9 to 9.2 ms (− 16 %). Vertical scale: sequence of stimuli applied. [file 42466_2023_293_MOESM2_ESM.tif]
