# Supplementary material for: Temporary and highly variable recovery of neuromuscular dysfunction by electrical stimulation in the follow-up of acute critical illness neuromyopathy: a pilot study
Source: Neurol Res Pract. 2023 Dec 28;5:66. doi: 10.1186/s42466-023-00293-1 (PMC10753844; doi:10.1186/s42466-023-00293-1)
Supplement: Supplementary file 3 — Additional file 3. Table S1 Normal values in motor nerve conduction studies at the institutional EMG Laboratory. [file 42466_2023_293_MOESM3_ESM.docx]

**Table S1:** Normal values in motor nerve conduction studies at the institutional EMG Laboratory

**Age range (yrs) minimal distal CMAP maximal distal minimal motor NCV**

**amplitudes ) motor latencies**

| **Peroneal nerve** |  |  |  |
| --- | --- | --- | --- |
| **40 - 60** | 3.2 mV | 5.2 ms | 41.3 m/s |
| **61 - 80** | 3.2 mV | 5.8 ms | 40.5 m/s |
| **Tibial nerve** |  |  |  |
| 40 - 60 | 3.9 mV | 5.8 ms | 41.0 m/s |
| 61 - 80 | 3.0 mV | 6.4 ms | 40.0 m/s |
| **Ulnar nerve** |  |  |  |
| 40 - 60 | 4.1 mV | 3.4 ms | 47.8 m/s |
| 61 - 80 | 4.0 mV | 3.5 ms | 45.0 m/s |
